# The Expression Level of ABCC6 Transporter in Colon Cancer Cells Correlates with the Activation of Different Intracellular Signaling Pathways

**DOI:** 10.3390/pathophysiology29020015

**Published:** 2022-05-12

**Authors:** Vittorio Abruzzese, Caecilia H. C. Sukowati, Claudio Tiribelli, Ilenia Matera, Angela Ostuni, Faustino Bisaccia

**Affiliations:** 1 Department of Sciences, University of Basilicata, 85100 Potenza, Italy; v.abruzz@hotmail.it (V.A.); ilenia.matera@unibas.it (I.M.); 2 Fondazione Italiana Fegato ONLUS, AREA Science Park Basovizza, 34149 Trieste, Italy; caecilia.sukowati@fegato.it (C.H.C.S.); ctliver@fegato.it (C.T.)

**Keywords:** ABCC6, colon cancer cell lines, purinergic signaling, Quercetin, Probenecid, cell migration, PI3K/AKT signaling

## Abstract

The ATP-binding cassette sub-family C member 6 transporter (ABCC6) is mainly found in the basolateral plasma membrane of hepatic and kidney cells. In hepatocarcinoma HepG2 cells, ABCC6 was involved in cell migration. In the present study, we investigated the role of ABCC6 in colon cancer evaluating the effect of Quercetin and Probenecid, inhibitors of the ectonucleotidase NT5E and ABCC6, respectively, on migration rate of Caco2 and HT29 cell lines. Both drugs reduced cell migration analyzed by scratch test. Gene and protein expression were evaluated by quantitative reverse-transcription PCR (RT-qPCR) and Western blot, respectively. In Caco2 cells, in which ABCC6 is significantly expressed, the addition of ATP restored motility, suggesting the involvement of P2 receptors. Contrary to HT29 cells, where the expression of ABCC6 is negligible but remarkable to the level of NT5E, no effect of ATP addition was detected, suggesting a main role on their migration by the phosphatidylinositol 3′-kinase (PI3K)/Akt system. Therefore, in some colon cancers in which ABCC6 is overexpressed, it may have a primary role in controlling the extracellular purinergic system by feeding it with ATP, thus representing a potential target for a therapy aimed at mitigating invasiveness of those type of cancers.

## 1. Introduction

The ATP-binding cassette sub-family C member 6 transporter (ABCC6) is an ATP-dependent transporter mainly found in the basolateral plasma membrane of the liver and kidney. Mutations in the *ABCC6* gene are associated with pseudoxanthoma elasticum (PXE), an autosomal recessive disease characterized by a progressive ectopic calcification of elastic fibers in dermal, ocular, and vascular tissues [1,2,3,4]. Although the structural features of the protein have been quite characterized [5,6,7,8,9], a lot of evidence suggests that, apart from directly supplying the extracellular matrix with PPi, ABCC6 may be involved in other specific functions, such as cholesterol homeostasis [10,11].

Control of ABCC6 expression has also been studied and seems to be finely regulated. The promotor region revealed two evolutionary conserved sequence blocks, the first of which is under epigenetic control, since DNA methylation of the CpG islands are inversely correlated with ABCC6 expression in a cell-type-specific manner [12]. The promotor region also contains several transcriptional-factor binding sites such as for agonists of the retinoid X receptor (RXR) [13], for pleomorphic adenoma gene (PLAG) transcription factors [14] and for the zinc finger transcription factor specificity protein 1 (SP1), which may be responsible for TGF-β1-induced expression of ABCC6 and other genes [15]. Finally, a crucial role in regulation of ABCC6 transcription is played by the hepatocyte nuclear factor 4 alpha (HNF4a) [16]. The activity of this transcription factor, which imprints a tissue-specific expression, can also be modulated through the interaction with other proteins or pathways, such as ERK cascade or the CCAAT/Enhancer binding protein ß (C/EBPß), which reduces or increases HNF4α binding to the promotor of ABCC6, thus ultimately regulating its expression [17,18].

Studies have revealed that the overexpression of ABCC6 in HEK293 cells results in the cellular efflux of ATP and other nucleoside triphosphates [19,20]. In the extracellular environment, ATP can be hydrolyzed into AMP by ENPP1 (ectonucleotide pyrophosphatase/phosphodiesterase 1) and CD39 (ectonucleoside triphosphate diphosphohydrolase-1, NTPDase-1); in turn, AMP is dephosphorylated into adenosine by CD73 (ecto-5′-nucleotidase, Ecto5′NTase), a key regulator in some tumor processes such as invasion, migration, and metastasis [21,22]. Since PPi is an inhibitor of mineralization, it was proposed that the absence of circulating PPi in PXE patients results in the ectopic mineralization of soft tissues, a typical feature of PXE. In addition to supplying the extracellular environment with PPi, ABCC6 contributes to the extracellular adenosine pool. While ATP and ADP play important roles as extracellular signaling molecules acting on the P2X and P2Y receptors, adenosine activates P1 receptors. Both families of purinergic receptors could have a role in regulation of key basic cellular functions. Several studies have provided interesting evidence about the involvement of purine nucleotides in neoplasia onset and progression [23,24]. Our previous studies showed that *ABCC6* knockdown, as well as its pharmacological inhibition by Probenecid, led to a reorganization of the cytoskeleton and downregulated the expression of CD73. The administration of adenosine or ATP restored the normal architecture of filopodia and migration rate, thus indicating that this effect was most likely due to a reduction in the extracellular ATP pool, through a modulation of ABCC6-mediated ATP efflux [25,26]. Quercetin decreased the expression of ABCC6 through the regulation of the AKT signaling pathway, thus also contributing to cytoskeleton rearrangement and reduced cell motility [27].

There is little information on the diagnostic or prognostic role of ABCC6 in some types of cancer [28,29,30,31], as well as, to the best of our knowledge, on the role of ABCC6 in colorectal cancer. According to Global Cancer Statistics 2020 [32], colorectal cancer ranks third in terms of incidence (10%) and second in terms of mortality (9.4%) in both sexes. In order to identify new biomarkers and to suggest new therapeutic approaches, for colorectal cancer, we studied the role of ABCC6 in colon cancer cell lines Caco2 and HT29. It has been reported that the ABCC6 gene was downregulated in colon adenocarcinoma, compared to control tissues [33]. Moreover, ABCC6 expression was found to be higher in human colon cancer-derived cell line Caco2, compared to normal intestine [34]. Involvement of ABCC6 in drug resistance in this type of cancer has been also reported, to some degree [35,36].

Extensive literature has been produced about the extracellular purinergic system in colon cancer. Nylund et al. have provided considerable evidence on the expression of functional ATP receptors in human colon adenocarcinoma-derived HT29 cells [37,38]. They also showed how members of the P2Y subfamily of receptors were significantly overexpressed in histological specimens from colon cancer patients, compared to tumor-free tissues [39]. It has been recently reported that ATP can promote invasion and migration in colon cancer cells and expression of epithelial–mesenchymal transition (EMT) related genes and tumor growth in vivo through the activation of the P2X7 receptor [40]. The effect of ATP seems to be dependent on concentration, since higher levels of ATP may inhibit proliferation and induce apoptosis, reflecting the activation of different receptors with lower affinity [41]. This could also explain the contrasting results disclosed by other researchers, who also found an increase in cell death and reduction in proliferation following treatment of high concentrations of ATP [42,43]. Finally, the presence of active nucleotide-metabolizing enzymes has been demonstrated in Caco2 and HT29 cells [44,45].

The aim of the present study was to investigate the role of ABCC6 in the modulation of both the extracellular purinergic system and phosphatidylinositol 3-kinase (PI3K)/Akt pathway in colon cancer-derived Caco2 and HT29 cells. The main results obtained show that both pathways are involved in cell motility and that ABCC6 substantially contributes to the activation and modulation of the purinergic system by a coordinated expression with the NT5E/CD73 ectonucleotidase.

## 2. Materials and Methods

### 2.1. Cell Culture and Treatments

Colorectal adenocarcinoma cell lines Caco2 and HT29 were grown in high glucose (4.5 g/L) Dulbecco’s modified Eagle’s medium (DMEM), supplemented with 10% fetal bovine serum (FBS), 2 mM L- glutamine, 100 U/mL penicillin and 100 µg/mL streptomycin. Cell cultures were maintained at 37 °C in a water-saturated atmosphere with 5% CO_2_. Cells were treated when they were approximately at 70% confluence. Cells lines were kindly provided by Prof. G. Grassi (University of Trieste). Quercetin and Probenecid were dissolved in DMSO at a concentration of 20 or 30 mg/mL, respectively, as stock solution. ATP was dissolved in water at a concentration of 100 mg/mL as a stock solution. Final concentration of DMSO did not exceed 0.25% or 0.5%, depending on the experiment. All compounds were purchased from Sigma (Sigma, Saint Louis, MO, USA).

### 2.2. Viability Assay

The effect of test compounds on cell viability was assessed by MTT (3-(4, 5-dimethyl thiazol-2yl)-2, 5-diphenyl tetrazolium bromide) assay. Then, 50,000 cells/cm^2^ were seeded onto a 96-well plate and were allowed to grow for 24 h. Then, they were treated with progressive dilution of Quercetin or Probenecid for 24 or 48 h, and afterward, they were incubated with fresh medium containing 0.75 mg/mL MTT for 4 h at 37 °C. Once the medium was removed, a stop solution of 1:1 DMSO and isopropanol with 1% of Triton X-100 was added to dissolve the formazan crystals, and absorbance at 570 nm with blank subtraction at 630 nm was measured using a microplate reader (Multiskan TM GO Microplate Spectrophotometer, Thermo Scientific, Waltham, MA, USA). Viability of treated cells was determined by comparing absorbance values with those of control cells treated with vehicle only.

### 2.3. Migration Assay

The effect of the test compounds on cell migration was assessed by in vitro wound healing assay, as previous described [27]. Briefly, Caco2 and HT29 were seeded onto a 6-well plate and was allowed to grow until they formed a nearly confluent monolayer. Then, they were treated with Quercetin 165 μM or Probenecid 250 μM in the presence or absence of ATP 500 μM for 12 h, and a linear scratch was performed in the cell monolayer with a sterile 10 μL plastic pipette tip. Cells were treated for further 24 h with the same treatments in the culture medium with a reduced percentage of FBS (1%). Pictures of the scratch were taken at the time of the scratch and at the end of the experiment using a Nikon Eclipse TS 100 inverted microscope equipped with a Nikon Coolpix P6000 digital camera (objective magnification 10x) (Minato, Tokyo, Japan) and were processed with free software Icy image to measure the area of the scratch. Scratch area filled after observation time was calculated as the difference in the area of a representative portion of the scratch at time 0 and after 24 h (A_T0_-A_T24 h_).

### 2.4. Western Blot

Cell pellets were suspended in Laemmli sample buffer (60 mM Tris–HCl pH 6.8, 10% glycerol, 2% SDS, 1% 2-mercaptoethanol, and 0.002% bromophenol blue) supplemented with a cocktail of proteases and phosphatase inhibitors and were sonicated to lyse the cell membranes. Cell lysates were resolved by SDS-PAGE on an 8% gel and protein transferred onto a nitrocellulose membrane. After a 1 h blocking at RT with 5% nonfat dried milk in PBS or TBS with 0.05% Tween 20, membranes were probed overnight at 4 °C with primary antibodies: 1:1000 anti-β-actin (a2066, Sigma-Aldrich, Darmstadt, Germany), 1:50 anti-ABCC6 (MA1-26542, ThermoFisher 0, Waltham, MA, USA), 1:250 anti-CD73 (1D7, ThermoFisher Scientific), 1:1000 anti-α-tubulin (T9026, Sigma-Aldrich, Darmstadt, Germany), 1:1000 anti-AKT (pan) (C67E7, Cell Signaling, Danvers, MA, USA), 1:2000 anti-phospho-AKT (Ser 473) (D9E, Cell Signaling, Danvers, MA, USA). The membranes were washed three times with PBST or TBST and incubated with the appropriate horseradish peroxidase-conjugated secondary antibodies at room temperature for 1 h, and the signals were visualized by the ECL™ Western Blotting Detection Reagents (GE Healthcare, Chicago, IL, USA) or the SuperSignal™ West Pico PLUS Chemiluminescent Substrate (Thermo Scientific, Waltham, MA), using the Chemidoc TM XRS detection system equipped with Image Lab Software for image acquisition (BioRad, Hercules, CA, USA). Densitometric analysis was performed by using GelAnalyzer 2010 software (Debrecen, Hungary). Protein expression level in treated cells was expressed as a fold change of expression in control cells treated with vehicle only. p-AKT/AKT ratio was obtained by dividing fold change of phosphorylated form per that of total AKT.

### 2.5. Real-Time PCR

RNA was extracted from cell pellets by using the Quick-RNA MiniPrep kit (Zymo Research, Irvine, CA, USA) and was retrotranscripted to cDNA using random primers and the High-Capacity cDNA Reverse Transcription kit (Applied Biosystem, Waltham, MA, USA) as per producer’s protocol. cDNA was amplified using iTaqTM Universal SYBR Green Supermix (Bio-Rad, Waltham, MA, USA) with the 7500 Fast Real-Time PCR System (Applied Biosystems). Primers were designed to span exon–exon junctions to prevent unwanted genomic DNA amplification (Table 1).

Quantification of transcripts was obtained with comparative threshold cycle method (2^−^^ΔCt^) with β-actin as the endogenous reference control.

### 2.6. Statistical Analysis

All of the assays were performed at least three times independently. The standard error of the mean (sem) or the 95% confidence interval were reported as measures of variability. Statistical analysis was performed by one- or two-way ANOVA, followed by appropriate post hoc analysis, using GraphPad Prism software (GraphPad Software, Inc., La Jolla, CA, USA). A *p* value < 0.05 was used as the minimum level of significance.

## 3. Results

### 3.1. Effect of Quercetin and Probenecid on Cells Viability

**Figure 1:**
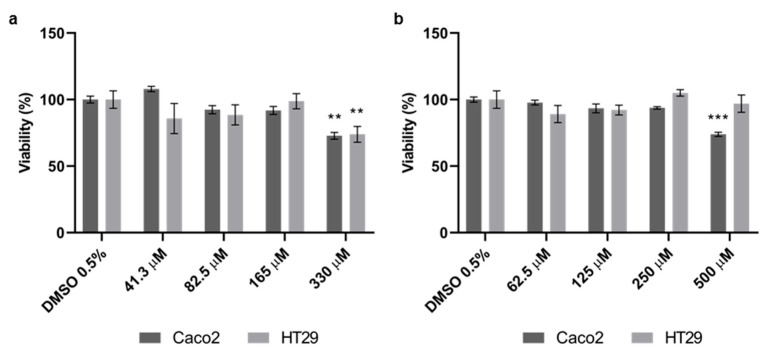
Effects of (**a**) Quercetin and (**b**) Probenecid on Caco2 and HT29 cell viability. Viability of treated cells was expressed as a percentage of the control group treated with vehicle alone (DMSO 0.5%) and are presented as the mean ± sem of at least three independent experiments. Statistical analysis was performed by two-way ANOVA followed by Dunnett correction for multiple comparisons. ** *p* < 0.01; *** *p* < 0.001.

In order to assess the effect of Quercetin and Probenecid on cell viability, an MTT assay was performed on Caco2 and HT29 cells. We treated the cells with various concentrations of test molecules for 24 h, and we obtained the effects on cell viability by comparing MTT reduction in cells treated with drugs or vehicle only (Figure 1). 

We did not observe any toxic effects up to a concentration of 165 μM for Quercetin or 250 µM for Probenecid. Therefore, the highest non-toxic concentrations of both Quercetin and Probenecid were chosen for the following experiments.

### 3.2. Effect of Quercetin and Probenecid on ABCC6 Expression

**Figure 2:**
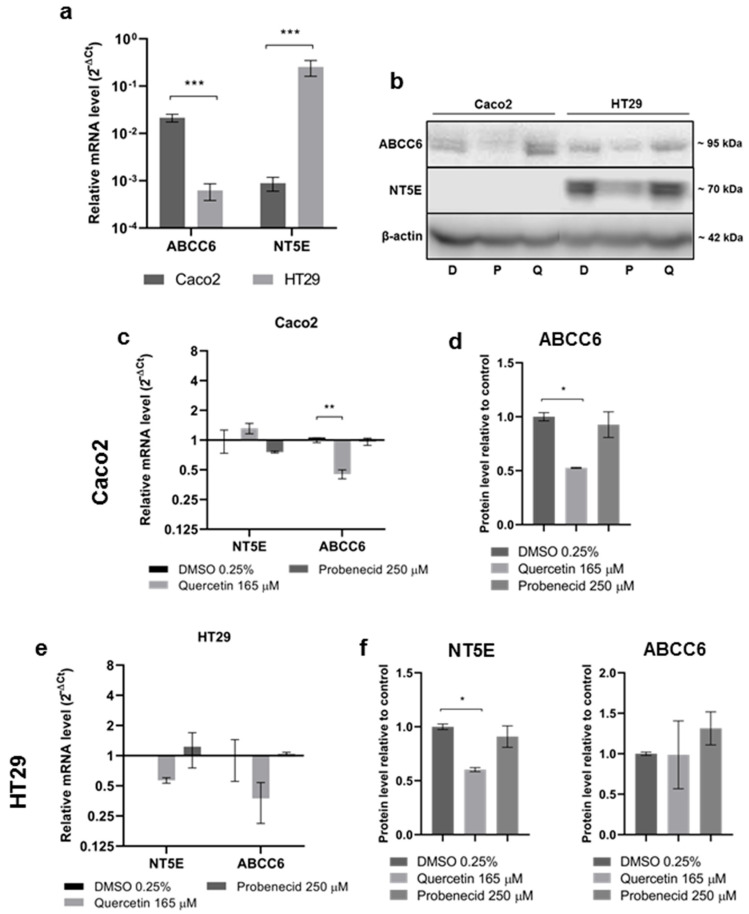
(**a**) Relative mRNA expression levels of ABCC6 and NT5E gene in Caco2 and HT29 cells. Results are expressed as the 2^−ΔCt^ and are presented as the mean ± sem of at least three independent experiments. β-actin was used as a reference gene. Statistical analysis was performed by 2-way ANOVA followed by Holm–Sidak correction for multiple comparisons. *** *p* < 0.001. (**b**) Representative Western blot of the effect of Quercetin and Probenecid on protein expression (D, DMSO 0.25%; Q, Quercetin 165 μM; *p*, Probenecid 250 μM). mRNA and protein expression levels of NT5E and ABCC6 in Caco2 (**c**,**d**) and HT29 (**e**,**f**) cells. Result are expressed as the 2^−ΔCt^ and are presented as the mean ± sem of at least three independent experiments. β-actin was used as a reference gene. Statistical analysis was performed by 2-way ANOVA followed by Dunnett’s correction for multiple comparisons. * *p* < 0.05, ** *p* < 0.01. Cells treated with vehicle only (DMSO 0.25%) were used as a control, and β-actin was used as a loading control. Data are presented as the mean ± sem of three independent experiments. Statistical analysis was performed by one-way ANOVA followed by Dunnett’s correction for multiple comparisons. * *p* < 0.05.

In this set of experiments, we tested the effect of Quercetin and Probenecid on ABCC6 expression, involved in efflux of ATP from cells. Moreover, as in hepatoma cells, we found a correlation between ABCC6 and NT5E expression, the ectonucleotidase responsible for the production of adenosine, and we assessed basal level of expression of both genes. ABCC6 was about 25 times more expressed than NT5E in Caco2; NT5E expression was about 400 times higher than ABCC6 in HT29 cells (Figure 2a). In Caco2 cells, NT5E protein expression was not detected by primary monoclonal antibody, as no bands appeared in the Western blot (Figure 2b); both mRNA and protein expression of ABCC6 were significantly downregulated by Quercetin in Caco2 cells (Figure 2c,d). In HT29 cells, we found a relevant, yet not statistically significant reduction in both ABCC6 and NT5E transcripts induced by Quercetin (Figure 2e), while NT5E protein was significantly downregulated by Quercetin (Figure 2f).

### 3.3. Quercetin and Probenecid Affect the Cell Migration Rate: Role of PI3K/Akt and Purinergic Pathways

**Figure 3:**
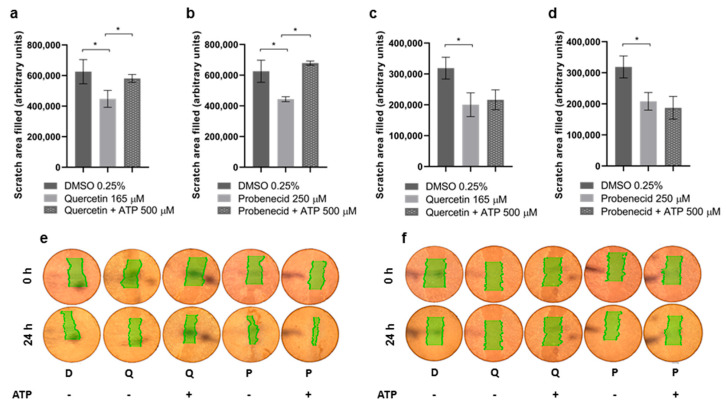
Effect of Quercetin and Probenecid on Caco2 and HT29 cell migration rate. Caco2 (**a**,**b**) and HT29 (**c**,**d**) cells were treated with a test molecule for 12 h. Then, a scratch was made in the cell monolayer, and pictures were taken after 24 h, while the cells were still kept in contact with Quercetin (Q) or Probenecid (P). DMSO-treated cells (D) were used as a control. ATP 500 μM was added to both Quercetin- and Probenecid-treated cells. Representative pictures of the scratches are shown in (**e**,**f**) for Caco2 and HT29 cells, respectively. Scratch area filled after observation time (difference in the scratch area, highlighted in green, at time 0 and after 24 h) was expressed as the mean and the standard error of at least three replicates. Statistical significance was assessed by one-way ANOVA followed by Holm–Sidak correction for multiple comparisons. * *p* < 0.01.

To study the effects of Quercetin and Probenecid on collective migration in Caco2 and HT29 cell lines, an in vitro scratch test was performed. As shown in Figure 3, both drugs markedly reduced the migration rate in the two cell lines. However, while in Caco2 cells the addition of ATP restored motility, no effect was detected on HT29 cells. 

**Figure 4:**
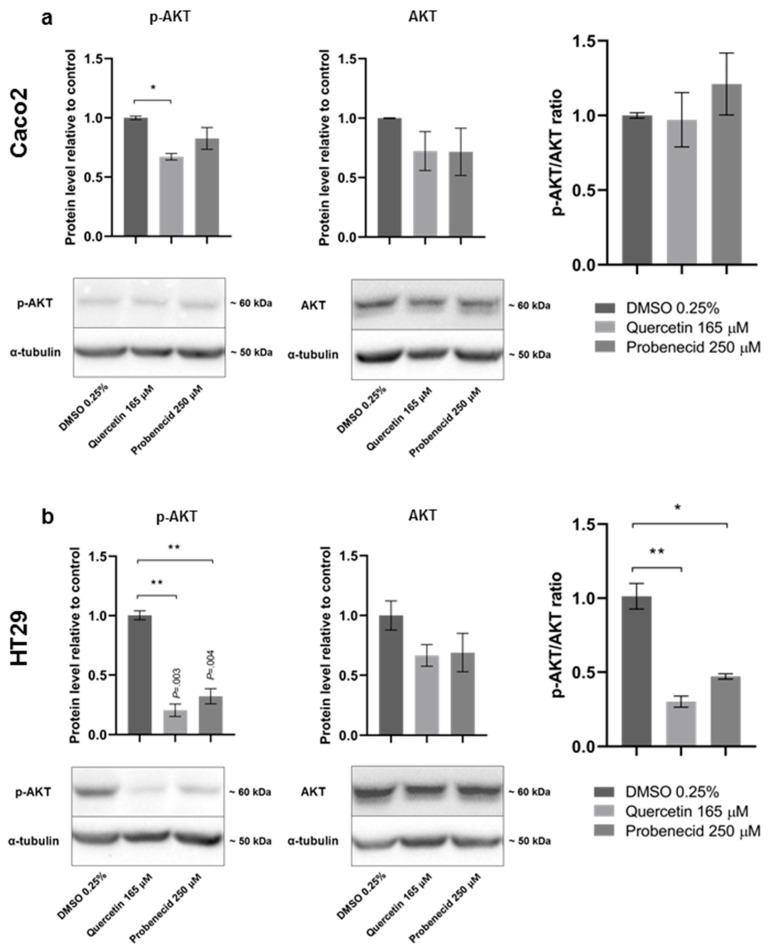
Effects of Quercetin and Probenecid treatment on AKT phosphorylation in (**a**) Caco2 and (**b**) HT29 cells. Cells treated with vehicle only (DMSO 0.25%) were used as a control. α-tubulin was used as a loading control. Data are presented as the mean ± sem of three independent experiments. Statistical analysis was performed by one-way ANOVA followed by Dunnett correction for multiple comparisons. * *p* < 0.05; ** *p* < 0.01.

We tested the effects of both Quercetin and Probenecid on the modulation of the AKT pathway, which is among the main regulators of colon cancer cell motility. The results clearly show a significant reduction in the *p*-AKT/AKT ratio, consistent with a reduced activation of the AKT pathway in Quercetin- and Probenecid-treated HT29 cells; no effect was found in Caco2 cells (Figure 4).

**Figure 5:**
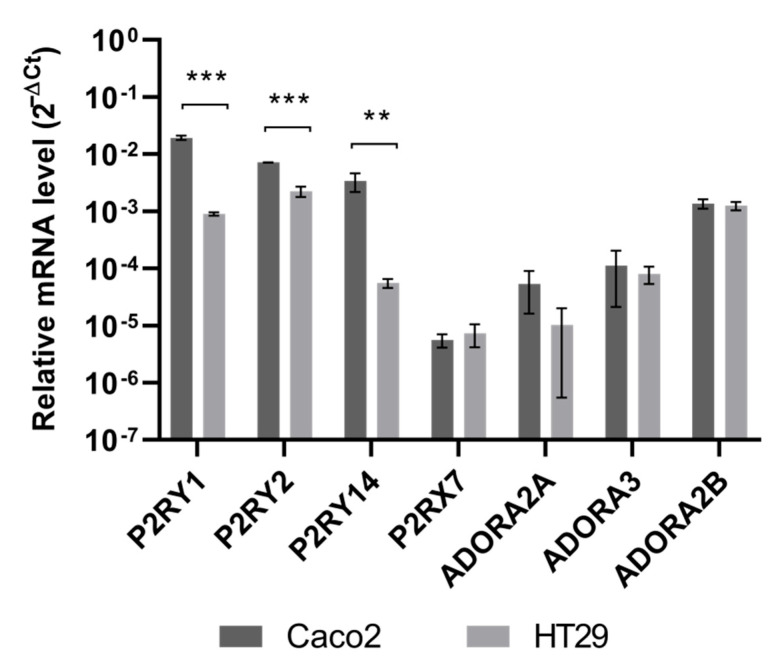
Purinergic receptor mRNA expression. Relative mRNA expression levels of some relevant purinergic receptors in Caco2 and HT29 cells. The results are expressed as the 2^−ΔCt^ and are presented as the mean and standard error of at least three replicates. β-actin was used as a reference gene. Statistical analysis was performed by two-way ANOVA followed by Holm–Sidak correction for multiple comparisons. ** *p* < 0.01; *** *p* < 0.001.

Finally, we assessed the expression level of some relevant purinergic receptors in both cell lines and found the biggest difference in mRNA levels of three ATP receptors, P2RY1, P2RY2 and P2RY14, whose expression was significantly higher in Caco2, compared to HT29 cells (Figure 5).

Compared with HT29, Caco2 cells showed a higher expression level of P2 receptors. No significant differences in the expression level of some P1 receptors were observed.

## 4. Discussion

Studies performed by ours and other groups pointed out that ABCC6 may have different roles due to its activity of ATP efflux transporter [46]. Besides supplying the extracellular matrix with PPi, a strong inhibitor of mineralization, the transported ATP can contribute to the activation of the extracellular purinergic system, thus affecting cell migration and cytoskeleton rearrangement in cell lines derived from liver cancer, in which ABCC6 is normally expressed. Apart from the liver, it has also been reported that ABCC6 may be overexpressed in some extrahepatic tumors, where it can contribute to drug resistance, even if its role has been debated and is often considered marginal, compared to other ABC transporters. 

In this study, we investigated the role of ABCC6 in two colon cancer cell lines, which exhibit different ABCC6 expressions. Migration was investigated, and activation of intracellular pathways have been suggested. 

We found that ABCC6 was highly expressed in Caco2 cells and was almost absent in HT29 cells; the opposite was for NT5E, the rate-limiting enzyme in extracellular ATP metabolism. It is known that both cell lines express functional purinergic receptors and ectonucleotidases activity, thus suggesting that purinergic signaling could have a key role in controlling some important cancer aspects, such as proliferation, apoptosis and invasion [37,38,39,40,41,42,43]. Considered together, the different levels of components of the purinergic system of these two cell types suggest possible different pharmacological responses. We evaluated the effect on Caco2 and HT29 cells of Probenecid and Quercetin, compounds that inhibit the ectonucleotidases NT5E and ABCC6, respectively.

To the best of our knowledge, no effect of Probenecid has been evaluated on colorectal tumor; on the contrary, Quercetin anticancer properties have been investigated in a number of in vivo and in vitro studies that have proposed the involvement of different signaling pathways such as PI3K, MAPK and toll-like receptor 4/NF-κB [47]. 

**Figure 6:**
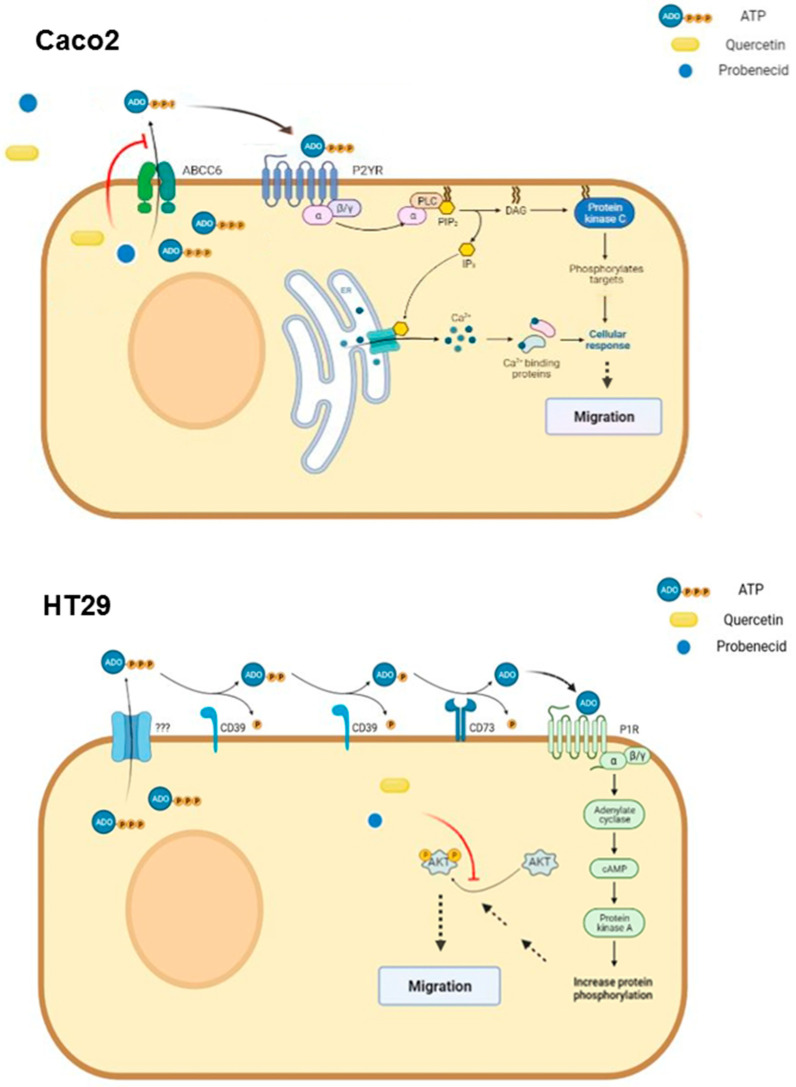
Proposed alternative pathways controlling colon cancer cell migration: role of ABCC6. In Caco2 cells (upper panel), ABCC6-secreted ATP activates P2Y receptors, causing an intracellular signaling cascade that controls cell migration. Quercetin and Probenecid reduce the extracellular ATP pool by inhibiting ABCC6 transport activity. In HT29 cells (lower panel), ATP is extruded through unspecified transporters and is converted into adenosine by ectonucleotidase enzymes CD39 and CD73. Activation of adenosine receptors triggers intracellular pathways leading to phosphorylation of AKT, which ultimately controls cell migration. Quercetin and Probenecid act downstream, reducing AKT phosphorylation through an unspecified mechanism. Quercetin also inhibits CD73 activity.

Significant reductions in migration rate were observed in both cell types treated with either Probenecid or Quercetin. The restoration of migration rate in Caco2 cells following ATP addition suggests that, in these cells, migration may be dependent on extracellular ATP signaling. At the same time, our hypothesis that the extracellular ATP pool is controlled by ABCC6 is based on the evidence that Probenecid is an inhibitor of ABCC6 transport activity, while Quercetin downregulates its expression. As reported, Quercetin is an inhibitor of both HNF4 [48] and C/EBPβ [49], two key transcriptional regulators of ABCC6 [16,17,18]. As a consequence, both treatments might modulate the purinergic signal through the involvement of P2 receptors for purine nucleotides, which have been found to be significantly expressed in Caco2 compared to that of HT29. It is known that in Caco2 cells, activation of P2Y receptors triggers mitogen-activated protein kinases cascade, leading to the phosphorylation of extracellular signal-regulated kinases 1 and 2 (ERK) and recruitment of downstream effectors [50,51], which can ultimately affect cell migration by controlling EMT-related gene expression [52] (Figure 6, upper panel). 

In HT29 cells, where the expression of ABCC6 is negligible, the activation of the purinergic signal can be controlled by some auxiliary players. The overexpression of NT5E in these cells suggests a role of adenosine P1 receptors, which through the phosphatidylinositol 3′-kinase (PI3K)/Akt system, may have a predominant role on their migration [53] (Figure 6, lower panel). The importance of this cell pathway in colon cancer progression has been clearly shown, since its downstream effector, the Mammalian Target of Rapamycin complexes 1 and 2 (mTOR1/2), are often overexpressed in advanced stages of colon cancer [54]. Their inhibition has been proposed as a strategy to mitigate invasion and migration of colon cancer cells, being involved in the regulation of EMT and cytoskeleton rearrangement, via RhoA and Rac1 signaling, which are members of the GTPases family that regulates actin filament assembly [55]. Many authors have reported that downregulation of the PI3K/AKT/mTOR pathway effectively inhibits migration in HT29 cells [56,57]. In HT29 cells, we found that Quercetin and Probenecid significantly reduced phosphorylation of AKT, which can explain an effect on cell motility independently from the involvement of P2 receptors. In both HT29 and HepG2 cells, the addition of ATP to the culture medium, together with Quercetin, did not restore the migration rate [27]. The inhibition of the migration rate induced by Probenecid in HT29 (not restored by ATP) and the decrease in the pAKT/AKT ratio might suggest some effect of Probenecid on this system as reported by different other studies [58,59].

In conclusion, the model we used in this work is particularly emblematic, since in Caco2 cells, in which ABCC6 is highly expressed, it may have a major role in controlling cell migration by feeding the extracellular purine pool with ATP. On the contrary, in HT29 cells, which are lacking ABCC6 expression, but show remarkable levels of NT5E expression, and probably higher ectonucleotidase activity, adenosine could have a predominant role on PI3K/AKT activation accordingly with what has been previously reported. 

## Figures and Tables

**Table 1:** Primers used in reverse transcription–quantitative polymerase chain reaction assay.

Gene	Primer Forward	Primer Reverse
ACTB	5′-CCTGGCACCCAGCACAAT-3′	5′-GCCGATCCACACGGAGTACT-3′
ABCC6	5′-ATCACTGATCCTTCCATCTTG-3′	5′-ACCAGCGACACAGAGAAGAGG-3′
NT5E	5′-GGGCGGAAGGTTCCTGTAG-3′	5′-GAGGAGCCATCCAGATAGACA-3′
P2RY1	5′-TGTGGTGGTGGCGATCTCC-3′	5′-TCGCAGGTACTCGTCTGAGG-3′
P2RY2	5′-TCAGCATTGTGTTCTTTGGG-3′	5′-CTGGGAAATCTCAAGGACTG-3′
P2RY14	5′-TCAGCATTGTGTTCTTTGGG-3′	5′-TGCTGTAACTCACTGACTGG-3′
ADORA2A	5′-AACCTGCAGAACGTCACCAA-3′	5′- GTCACCAAGCCATTGTACCG -3′
ADORA3	5′-GTCAGATACAAGAGGGTCAC-3′	5′-GTCAGTTTCATGTTCCAGCC-3′
ADORA2B	5′-GAGACACAGGACGCGCTGTACG-3′	5′-CGGGTCCCCGTGACCAAACT-3′

ACTB, actin beta; ABCC6, ATP binding cassette subfamily C member 6; NT5E, 5′-Nucleotidase Ecto; P2RY1, purinergic receptor P2Y1; P2RY2, purinergic receptor P2Y2; P2RY14, purinergic receptor P2Y14; ADORA2A, adenosine A2a receptor; ADORA3, adenosine A3 receptor, ADORA2B, adenosine A2b receptor.

## Data Availability

Not applicable.
